# Efficacy of a higher-flexibility duodenal stent for palliation of gastric outlet obstruction

**DOI:** 10.1055/a-2539-9270

**Published:** 2025-03-14

**Authors:** Noa Eveline Adriana Kapteijn, Pauline A. Zellenrath, Peter Siersema, Agnes N Reijm, Lydi Van Driel, Pieter J.F. de Jonge, Wim J. Lammers, Judith Honing, Manon C.W. Spaander

**Affiliations:** 1 6993 Gastroenterology and Hepatology, Erasmus MC University Medical Center Rotterdam, Rotterdam, Netherlands; 2 84744 Gastroenterology and Hepatology, Reinier de Graaf Gasthuis, Rotterdam, Netherlands

**Keywords:** Endoscopy Upper GI Tract, Reflux disease, Malignant strictures, Dilation, injection, stenting, Quality and logistical aspects, Performance and complications

## Abstract

**Background and study aims:**

Duodenal self-expandable metallic stent (SEMS) placement is a common palliative treatment for malignant gastric outlet obstruction symptoms (GOOS). The higher flexibility of the WallFlex Duodenal Soft stent aims to ease stent placement and reduce adverse events (AE). This descriptive study compared the WallFlex Soft stent with other duodenal SEMS designs with regard to efficacy and safety.

**Patients and methods:**

Patients receiving the WallFlex Duodenal Soft stent as first-line treatment for GOOS were included in a prospective cohort (2019–2023). A retrospective cohort (1998–2019) with patients treated with other duodenal SEMS designs as first-line treatment for GOOS was used for comparison.

**Results:**

In the prospective cohort, 11 patients were treated with the WallFlex Duodenal Soft, achieving 100% technical and 82% clinical success rates. The retrospective cohort of 147 patients with various types of duodenal SEMS showed 97% technical and 86% clinical success. For the WallFlex Duodenal Soft vs. the other duodenal SEMS, the overall survival was 101 vs. 82 days and median symptom-free survival was 83 vs. 28 days. AE rates were 18% vs. 28%, respectively.

**Conclusions:**

The WallFlex Duodenal Soft stent effectively treats GOOS in palliative patients and seems to be associated with longer symptom-free survival and lower AE rates compared with previous duodenal SEMS designs.

## Introduction

Malignant gastric outlet obstruction (GOO) is a late complication of advanced gastric and periampullary malignancies that causes distressing symptoms including abdominal pain, nausea, vomiting, and weight loss 1 2 . These symptoms can drastically diminish patient quality of life, necessitating prompt and effective management. Traditional palliative management involves surgical intervention with gastrojejunostomy (GJJ), but this technique is associated with high morbidity and mortality rates and is often precluded by tumor size and location or patient health status 1 2 3 . Less invasive endoscopic techniques, such as duodenal self-expandable metallic stent (SEMS) placement and more recently, endoscopic ultrasound (EUS)-guided gastrojejunostomy (GJJ), have emerged as effective alternatives 1 2 3 4 5 . Despite growing interest in EUS-guided GJJ, its reliance on highly skilled endoscopists and its relatively high costs make this technique less practical. In addition, SEMS treatment can often be performed on a relatively short-term basis for patients with a poor prognosis. Therefore, the ability to place a SEMS is imperative for patients in whom EUS-guided or surgical GJJ is not feasible.

Different stent designs are available for palliation of GOO symptoms 4 6 7 8 . In patients with expected short-term survival, uncovered SEMS are preferred over covered SEMS because they are less prone to migration 3 5 9 . Recently, the uncovered WallFlex Duodenal Soft stent (Boston Scientific, Marlborough, Massachusetts, United States) was developed 3 5 9 . This SEMS has greater flexibility and conformability due to both a lower axial and radial force than previous uncovered duodenal SEMS, including the original WallFlex Duodenal stent ( **Supplementary Table 1** ) 3 5 9 . This enhanced flexibility aims to decrease complications such as stent kinking, migration, perforation, and bleeding.

This descriptive study evaluated the efficacy and safety of the WallFlex Duodenal Soft Stent in alleviating symptoms of GOO among a prospective cohort of patients with malignant GOO. It compared these outcomes to various other duodenal SEMS designs included in a large retrospective cohort.

## Patients and methods

### Patients

For the prospective cohort, patients with malignant GOO symptoms eligible for duodenal stent placement were included ( Table 1 ) between December 2019 and September 2023. For comparison we used our retrospective cohort, including patients with malignant GOO symptoms treated with a duodenal stent at the Erasmus Medical Center between January 1998 and November 2019 ( Table 1 ) 7 .

**Table:** **Table 1** Baseline characteristics of patients in whom duodenal SEMS placement was successfully inserted in both cohorts.

	Prospective cohort 2019–2023 (n = 11)	Retrospective cohort 1998–2019 (n = 147)
Age, mean (SD), years	62 (14)	64 (12)
Sex, male, n (%)	8 (73)	91 (62)
PS at baseline, median (IQR)	2 (1–3)	–
GOOSS at baseline, median (IQR)	0 (0–1)	–
NRS for pain at baseline, median (IQR)	2 (0–4)	–
Tumor etiology, n (%)		
Pancreas	5 (45)	75 (51)
Bile duct	0	18 (12)
Stomach	2 (18)	14 9.5)
Colorectal	0	6 (4)
Duodenum	0	6 (4)
Gallbladder	3 (27)	6 (4)
Other	1 (9)	22 (15)
Prior treatment n (%)		
None	6 (55)	98 (67)
Chemotherapy	4 (36)	43 (29)
Radiotherapy	0	1 (1)
Chemoradiotherapy	1 (9)	5 (3)
Concomitant chemotherapy n (%)	0	18 (12)
Peritoneal deposits, n (%)	9 (82)	36 (24)
Ascites, n (%)	2 (18)	25 (17)
Sedation, n (%)		
Conscious sedation	5 (45)	138 (94)
Propofol	6 (55)	4 (3)
General anesthesia	0	1 (1)
SEMS, n (%)		
WallFlex	11 (100)	55 (37)
Evolution	0	68 (46)
Niti-Sl	0	10 (7)
Shu	0	1 (1)
UltraFlex	0	1 (1)
Unknown	0	8 (5)
Location stricture, n (%)		
Distal stomach	0	32 (22)
D1	7 (64)	67 (46)
D2	5 (45)	84 (57)
D3	3 (27)	31 (21)
D4	2 (18)	4 (3)
Unknown	1 (9)	0
Extent, stricture, n (%)		
Single compartment	3 (27)	73 (50)
Multiple compartments	9 (82)	70 (48)
Papilla covered, n (%)	3 (27)	36 (24)
GOOSS, gastric outlet obstruction scoring system; IQR, interquartile range; NRS, numeric rating scale; PS, performance status; SD, standard deviation; SEMS, self-expandable metallic stent.		

### Inclusion and exclusion criteria

Both cohorts included patients older than 18 years who experienced GOO symptoms from a malignant obstruction located in the distal stomach or duodenum, signed informed consent, and had at least 1 follow-up endoscopy. Patients who underwent previous palliative treatment for GOO symptoms were excluded from this study.

### Endoscopic procedure

**Fig. 1:**
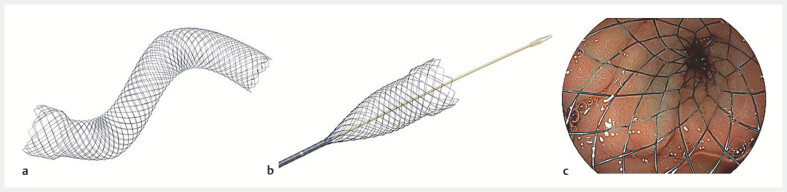
Design and placement of the WallFlex Duodenal Soft Stent. **a** WallFlex Soft duodenum stent. **b** Standard through-the-scope, over-the-wire placement technique. c Deployment of the WallFlex Soft duodenum stent in a patient. ( **a** and **b** courtesy of Boston Scientific.)

During the endoscopic procedure, patients had conscious or propofol sedation. The prospective study used only the WallFlex Soft stent ( Fig. 1 **a** ), whereas the retrospective cohort used various stents: Evolution (48%), Niti-S (7%), Shu (1%), UltraFlex (1%), WallFlex (39%), and unknown (6%). From 1998 to 2009, the WallFlex stent was predominantly used (72%), switching to the Evolution stent from 2010 to 2019 (91%). Placement involved a standard through-the-scope, over-the-wire technique ( Fig. 1 **b** ). In both cohorts, endoscopist judgment and preference determined stent length. For strictures measuring less than 4 cm, a 6-cm SEMS was used, whereas a 9- or 12-cm stent was chosen for strictures larger than 4 cm. In the prospective cohort, telephone interviews were used to assess the Gastric Outlet Obstruction Scoring System (GOOSS) (0 no oral intake, 1 liquids only, 2 soft solids only and 3 low-residue or full diet), and World Health Organization (WHO) performance status (PS), numeric rating scale (NRS) for pain assessment (0 no pain-10 severe pain), medication usage, and adverse events (AEs) were recorded. When an AE occurred, the cause and date of first onset were recorded. In addition, the cause and time of death for all patients were documented. Endoscopy reports provided data on stent placement and other interventions. In the retrospective cohort, endoscopy reports were used to retrieve data about initial duodenal stent placement and subsequent procedures 6 . Clinical success was defined as resolution of GOO symptoms, and technical success as proper stent deployment.

### Statistics

Baseline characteristics and outcomes, including technical and clinical success rates, were reported with descriptive statistical analysis. Categorical variables were presented as absolute or relative frequencies (%) and continuous variables as medians with range or interquartile range (IQR). Analyses were performed in IBM SPSS v.28.

## Results

### Patient characteristics

Between December 2019 and March 2023, 14 patients with GOO symptoms were eligible for palliative stent treatment, of whom 11 patients were enrolled in the prospective cohort (median age: 62 years, 73% male) ( **Supplementary Table 2** ). The remaining three patients did not sign informed consent or underwent EUS-guided GJJ. Pancreatic cancer was the most prevalent cancer (45%), followed by stomach cancer (18%) and hilar cholangiocarcinoma (18%). Malignant stenosis was predominantly located in the first part of the duodenum (D1-D2) (73%) and less than 4 cm in length (73%). Baseline PS was 2 (IQR 2–3), GOOSS was 0 (IQR 0–1), and pain was present in eight of 11 patients with a median NRS score of 2 (0–4). At baseline, seven patients used pain medication, primarily paracetamol (6/7), oxycodone (5/7), and/or fentanyl (2/7).

In the retrospective cohort, 147 patients underwent palliative stent treatment for GOO symptoms between January 1998 and November 2019 (median age: 64 years, 62% male) of whom 21% (n = 40) were excluded due to incomplete follow-up, stricture location not in the distal stomach or duodenum, use of a esophageal stent, prior GJJ, presence of a second stenosis, or missing endoscopy reports 6 . Pancreatic cancer was the most prevalent primary tumor etiology (51%), followed by bile duct tumors (18%). Stricture location and stent length were consistent across the prospective and the retrospective cohort. Obstructions were primarily located in the first or second part of the duodenum (64% and 45% vs. 46% and 57%) and less than 4 cm long (73% vs. 65%).

### Technical and clinical success rate

In the prospective cohort, the WallFlex Soft duodenal stent achieved 100% technical success in all 11 patients, effectively covering the stenosis ( Fig. 1 **c** ). In the retrospective cohort, technical success was achieved in 143 of 147 patients (97%). Clinical success in the prospective cohort was 82%, with a median overall survival of 101 days (range 9–421). The retrospective cohort had an initial clinical success rate of 86%, with a median survival of 82 days (range 1–448). Median GOOSS improved from 0 (IQR 0–1) to 2 (IQR 2–3) in 2 weeks in the prospective cohort, whereas median PS did not change, 2 (IQR 1–3) to 2 (IQR 1–2). In all patients in the prospective cohort, PS scores deteriorated approximately 1 to 3 weeks before death. GOO symptom recurrence occurred in 91% of patients after a median of 83 days (range 0–317), whereas in the retrospective cohort, this occurred in 57% of patients after a median of 28 days. Detailed outcomes are provided in Table 2 .

**Table:** **Table 2** Outcomes of palliative GOO duodenal SEMS treatment in both cohorts.

	Prospective cohort 2019–2023 (n = 11)	Retrospective cohort 1998–2019 (n = 147)
Clinical success rate, %	82	86
Technical success rate, %	100	97
Patients with recurrent GOO, n (%)	10 (91)	82 (59)
Overall survival (days), median (range)	101 (9–421)	82 (1–448)
GOO free survival (days), median (range)	83 (0–317)	28 (4–287)
PS after 2 weeks, median (IQR)	2 (1–2)	–
GOOSS after 2 weeks, median (IQR)	2 (2–3)	–
NRS for pain after 2 weeks, median (IQR)	0 (0–1)	–
Patients with ≥ 1 adverse event, n (%)	2 (18)	49 (33)
Adverse events		
Fever, n (%)	1 (9)	14 (10)
Cholangitis, n (%)	1 (9)	13 (9)
Hemorrhage, n (%)	0	3 (2)
Pressure necrosis, n (%)	0	2 (1)
Delirium, n (%)	0	1 (1)
Pancreatitis, n (%)	0	1 (1)
Perforation, n (%)	0	2 (1)
Pneumonia, n (%)	0	3 (2)
Stent length, n (%)		
6 cm	8 (73)	96 (65)
9 cm	2 (18)	34 (23)
12 cm	1 (9)	17 (12)
GOOSS, gastric outlet obstruction scoring system; IQR, interquartile range; NRS, numeric rating scale; PS, performance status; SEMS, self-expandable metallic stent.		

### Prior therapy and adverse events

Five patients (45%) in the prospective cohort had prior systemic therapy, with no impact on stent performance or AE rates. In the retrospective cohort, 67 patients (46%) had prior systemic therapy. Here, pre-stent therapy significantly affected stent performance and increased AE risk (OR 2.53, *P* = 0.02) in contrast to the prospective cohort. AEs occurred in two of 11 of the prospective and 40 of the 143 patients in the retrospective cohort (18% vs 28%). The prospective cohort reported two AEs, fever (9%) and cholangitis (9%), which were also the most common AEs in the retrospective cohort (10% and 9%) 6 . Post-stent pain scores were low, with a median NRS of 0 (IQR 0–1) in eight of 11 prospective patients. Additional AEs in the retrospective cohort included hemorrhage (2%), pressure necrosis (1%), delirium (1%), pancreatitis (1%), perforation (1%), and pneumonia (2%).

## Discussion

The WallFlex Duodenal Soft Stent demonstrated high technical and clinical success rates of 100% and 82%, respectively, which is similar to the other stent designs in the retrospective cohort [4 6,8,10–12]. However, the WallFlex Duodenal Soft stent seems to have longer overall and GOO symptom-free survival (101 vs. 82 and 83 vs. 28 days) and fewer stent-related AEs (18% vs. 28%). Patients in the prospective cohort had a relatively long median overall survival of 101 days (range 9–421 days), and a median GOO symptom-free survival of 83 days (range 0–317 days) after WallFlex Duodenal Soft stent placement. Comparable findings exist in studies involving the WallFlex stent versions 6 10 , whereas studies regarding other duodenal SEMS designs report a shorter median overall survival (76 days, range 62–82 days) and GOO symptom-free survival (18 days, range 4–28 days) [4,6 8]. The retrospective cohort reported shorter median survival times (82 and 28 days, respectively) compared with the prospective cohort, with equivalent distribution of tumor types and prior therapies. The improved GOO symptom-free survival in the prospective cohort can be attributed to flexibility of the WallFlex Duodenal Soft design, which could enhance anatomical fit and may improve efficacy 4 11 12 . In addition, the relatively good PS score of 2 at baseline in the prospective cohort may have affected outcomes. Although the GOO symptom-free survival was higher in the prospective cohort, 91% of patients eventually experienced a recurrence of GOO symptoms compared with 59% in the retrospective cohort. Patients in the prospective cohort experienced GOO symptoms recurrence 1 to 2 weeks before death. GOO symptom recurrence could be attributed to advanced disease stage accompanied by fast deterioration of PS and tumor progression. Interestingly, our results indicate that the WallFlex Soft stent may provide long-term efficacy, with a symptom-free survival of more than 300 days in two patients. This is in contrast to other duodenum SEMS studies, which reported shorter symptom-free survival periods 4 6 8 . It must be noted that the clinical success rate for the WallFlex Soft stent appears to depend on PS score of patients at baseline. Patients with lower PS scores (0 or 1) generally experienced improvement in optimal score and maintained favorable outcomes, whereas those with higher scores (3) did not. In our study, two patients with a PS score of 3 had no improvement in their GOOSS post-stent insertion. This indicates that stent effectiveness may be influenced by patient clinical condition; however, the small sample size restricts the robustness of these conclusions.

The flexible design of the WallFlex Soft stent also aims to mitigate risk of AEs such as stent kinking, migration, obstruction, perforation, or bleeding 5 . Remarkably, no such events occurred in our prospective cohort. However, two patients (18%) experienced stent dysfunction after 1 week due to tumor ingrowth, a risk more common with uncovered stents compared with covered ones (15.8% vs. 3.1%) 3 . Stent occlusions theoretically can be managed with additional therapy, but this was not feasible for these patients due to their poor condition. In comparing AE rates with the retrospective cohort, the prospective cohort showed a reduction in AEs and related symptoms such as pain (28% vs. 18%). Notably, in the retrospective cohort, AEs increased from 25% to 33% from 2010 to 2019, with stent-related pain rising from 13% to 20% and a higher frequency of multiple AEs per patient. More patients were pretreated with chemo-and/or radiotherapy from 2010 to 2019, which may have contributed to the higher risk and frequency of AEs. Furthermore, stent type differed between the two time periods. In the period before 2010, the WallFlex stent was predominantly used (72%), whereas from 2010 to 2019, the Evolution duodenal stent was more commonly used (91%). The most prevalent AEs observed in both cohorts were fever and cholangitis. Fever was primarily attributed to post-procedure cholangitis and localized infections at the stent site. In addition, cholangitis was primarily observed in patients with strictures located in the D2 region, which is likely caused by overlap of the papilla. Cholangitis was predominantly managed with broad-spectrum antibiotics followed by endoscopic retrograde cholangiopancreatography (ERCP); if ERCP was not possible, percutaneous transhepatic drainage was used. Pain scores after stent placement showed low NRS scores of 0 (IQR 0–1) in eight of 11 patients in the prospective cohort. Furthermore, in the retrospective cohort, multiple AEs often occurred in the same patient 6 . The reduced AE incidence may be attributed to the WallFlex Soft stent flexible design, which minimizes axial force on surrounding tissue 9 . Notably, 45% of patients in the prospective cohort had prior chemotherapy or chemoradiotherapy, a factor linked with increased risk of AEs, as observed in the retrospective cohort 6 . However, this was not evident in our prospective cohort 7 11 .

This study has several limitations. The small sample size in the prospective cohort compared with the larger retrospective cohort restricted comparisons and permitted only descriptive analysis without statistical analysis. Moreover, lack of PS data in the retrospective cohort hinders comparisons. In the retrospective cohort data, prophylactic antibiotics were not administered before the stent procedure, which could potentially lead to more favorable outcomes. In addition, in the retrospective cohort, various stent designs were used, which complicates direct comparisons. Also, the small number of patients limits generalizability and prevents drawing firm conclusions. Although flexible design of the stent may reduce AEs, the small cohort size warrants cautious interpretation. Further research with larger sample sizes is needed to validate these findings.

## Conclusions

In conclusion, the WallFlex Duodenal Soft Stent demonstrated technical and clinical success rates comparable to other duodenal SEMS designs, but with fewer stent-related AEs and long-term efficacy.

## Supplementary Material

Supplementary Material Zusatzmaterial
