# Supplementary material for: Efficacy of a higher-flexibility duodenal stent for palliation of gastric outlet obstruction
Source: Endosc Int Open. 2025 Mar 14;13:a25399270. doi: 10.1055/a-2539-9270 (PMC11922175; doi:10.1055/a-2539-9270)
Supplement: Supplementary file 1 — Supplementary Material [file 10-1055-a-2539-9270_25428659.pdf]

Thieme

Supplementary material

Table S1. *Characteristics of duodenum SEMS used in prospective and retrospective cohort.*

Stent	Structure	Radial Force	Axial Force	Conformability	Flexibility
WallFlex Soft	Braided, nitinol alloy, uncovered	Low	Low to moderate	High	High
WallFlex	Braided, nitinol alloy, uncovered	Moderate	Low to moderate	Moderate	Moderate to high
Evolution	Laser-cut, nitinol alloy, with partially covered options	Moderate	High	Moderate	Moderate
Niti-S	Braided, nitinol, partially covered	Moderate	Moderate	Moderate to high	Moderate
Shu	Braided, stainless steel, uncovered, primarily for esophageal use	High	High	Low	Low

Table S2. *Baseline characteristics of the 11 patients of the prospective cohort who received the WallFlex duodenum soft stent.*

Case	SEMS	Sex	Age (years)	Primary tumor etiology	Stricture location	Stricture (cm)	Peritoneal deposits	PS score	GOOSS score	NRS score
1	Yes	Male	68	Stomach	D1	>4	No	3	1	2
2	Yes	Male	81	Stomach	D1-D2	>4	Yes	2	0	2
3	Yes	Male	68	Pancreas	D1	<4	No	2	0	0
4	Yes	Female	61	Non-Small Cell Lung Cancer	D2-D3	<4	Yes	3	1	4
5	Yes	Male	50	Pancreas	D3-D4	<4	Yes	1	0	2
6	Yes	Female	65	Pancreas	Unknown	<4	Yes	3	3	0
7	Yes	Female	69	Galbladder	D1	<4	Yes	1	0	2
8	Yes	Male	77	Pancreas	D3-D4	>4	Yes	3	0	0
9	Yes	Male	66	Hilar cholangiocarci noma	D1-D2	<4	Yes	2	0	2
10	Yes	Male	33	Hilar cholangiocarci noma	D1-D2	<4	Yes	2	0	4
11	Yes	Male	44	Pancreas	D1-D2	<4	Yes	3	0	3
